# Supplementary material for: Mid-upper arm circumference as a simple tool for identifying central obesity and insulin resistance in type 2 diabetes
Source: PLoS One. 2020 May 21;15(5):e0231308. doi: 10.1371/journal.pone.0231308 (PMC7241705; doi:10.1371/journal.pone.0231308)
Supplement: S1 Table — (DOCX) [file pone.0231308.s001.docx]

Supplemental Table 1 [Hypoglycemic](javascript:;) agents

|  | DPP4Is | Metformin | TZDs | Glucosidase inhibitor |
| --- | --- | --- | --- | --- |
| Patients(N) | 21/103 | 76/103 | 12/103 | 38/103 |

DPP4Is: dipeptidyl peptidase 4 inhibitors; TZDs: Thiazolidinediones
