# Supplementary material for: Mid-upper arm circumference as a simple tool for identifying central obesity and insulin resistance in type 2 diabetes
Source: PLoS One. 2020 May 21;15(5):e0231308. doi: 10.1371/journal.pone.0231308 (PMC7241705; doi:10.1371/journal.pone.0231308)
Supplement: S2 Table — (DOCX) [file pone.0231308.s002.docx]

Supplemental Table 2 Hypotensive agents

|  | β-receptor blocker | Calcium Antagonists | Diuretics | ACEI/ARB | α-adrenergic blockers |
| --- | --- | --- | --- | --- | --- |
| Patients | 12/103 | 30/103 | 5/103 | 54/103 | 13/103 |

ACEI/ARB: Angiotensin converting enzyme inhibitors/ Angiotensin Receptor Blocker
