# Supplementary material for: Mid-upper arm circumference as a simple tool for identifying central obesity and insulin resistance in type 2 diabetes
Source: PLoS One. 2020 May 21;15(5):e0231308. doi: 10.1371/journal.pone.0231308 (PMC7241705; doi:10.1371/journal.pone.0231308)
Supplement: S3 Table — (DOCX) [file pone.0231308.s003.docx]

Supplemental Table 3 Lipid-lowering agents

|  | fenofibrate | statins |
| --- | --- | --- |
| Patients | 18/103 | 70/103 |
