# Supplementary material for: Mid-upper arm circumference as a simple tool for identifying central obesity and insulin resistance in type 2 diabetes
Source: PLoS One. 2020 May 21;15(5):e0231308. doi: 10.1371/journal.pone.0231308 (PMC7241705; doi:10.1371/journal.pone.0231308)
Supplement: S4 Table — (DOCX) [file pone.0231308.s004.docx]

Supplemental Table 4 Uric-acid-lowering agents

|  | Allopurinol | Benzbromarone | Febuxostat |
| --- | --- | --- | --- |
| Patients | 2/103 | 12/103 | 6/103 |
