# Supplementary material for: Mid-upper arm circumference as a simple tool for identifying central obesity and insulin resistance in type 2 diabetes
Source: PLoS One. 2020 May 21;15(5):e0231308. doi: 10.1371/journal.pone.0231308 (PMC7241705; doi:10.1371/journal.pone.0231308)
Supplement: Attachment — Submitted filename: Comments to Editor.docx [file pone.0231308.s005.docx]

**Comments to Editor**

Dear Dr. Mauro Lombardo,

Thanks for inviting me as the reviewer. I would like you to see below.

There was the previous article [Hou Y, et al. BMJ Open 2019; 9: e028904. doi:10.1136/bmjopen-2019-028904] . This article included 6287 participants with or without diabetes aged 40 years or older and was investigated from very various viewpoints. This article has already revealed almost all of this study’s results except the participants for not only T2DM (but including T2DM patients and investigated). Actually, I think this article did not have enough novelty even considering this study targeted only T2DM patients. Actually, I think this article should be rejected. If authors investigated and mentioned properly what I wrote perfectly, I would like to reconsider, so I chose “Major Revision”.

Please consider kindly.

Sincerely,

Ichiro Abe
