# Supplementary material for: Mid-upper arm circumference as a simple tool for identifying central obesity and insulin resistance in type 2 diabetes
Source: PLoS One. 2020 May 21;15(5):e0231308. doi: 10.1371/journal.pone.0231308 (PMC7241705; doi:10.1371/journal.pone.0231308)
Supplement: Attachment — Submitted filename: Supplemental file for review.docx [file pone.0231308.s006.docx]

**Point-by –Point Answers to**

**the Associate Editor’s and Reviewers’ comments**

**Reviewer #2: Dear author**s,

This article is interesting because authors investigated the importance MUAC. However I think this article has unavoidable problems to be confirmed.

**1、** Considering this study was performed for T2DM patients, authors should investigate the effect of T2DM itself to results in detail. Authors wrote “without insulin treatment or other medications that could alter insulin secretion (such as Sulphonylurea) for at least 2 weeks, not having severe disease and be free of any acute infection during 2 weeks before the inclusion” at MATERIALS and METHODS and patients’ HbA1c levels in Table 1. At first, authors should write details of agents for T2DM which patients were administered when they were investigated. For instance, almost all agents for T2DM affected to insulin profile, DPP4Is and/or SGLT2Is particularly (of course, glinide were not used, I think). If patients used these two agents in particular, it must affect the results (If patients did not use these two agents, authors should write detail of agents for T2DM in Table 1).

**Answer:**

Thank you for your comment.

We’ve added the details of hypoglycemic agents in the MATERIALS AND METHODS and supplemental table 1 as you suggested. All the patients were treated without insulin treatment, Sulphonylurea, sodium-dependent glucose transporters-2 inhibitors (SGLT-2i) or glucagon-like peptide-1 (GLP-1) analog for at least 2 weeks. (see Paragraph 2 in Page 5 of the resubmitted manuscript with tracked changes)

**2、**Indeed, authors should investigate the association between MUAC and glycemic parameters because authors targeted T2DM patients. If authors are thinking there is no necessity to consider glycemic parameters in this study, this study’s aim could not be understood by many of readers (including me) as they will not understand the difference between this study and the previous article. Moreover, authors must see the article, [Hou Y, et al. BMJ Open 2019; 9: e028904. doi:10.1136/bmjopen-2019-028904]. This article included 6287 participants with or without diabetes aged 40 years or older and was investigated from very various viewpoints. This article has already revealed almost all of this study’s results except the participants for not only T2DM. Actually, I think this article did not have enough novelty, and this study cohort was not large. So what I wrote above must be investigated and mentioned in order to gain novelty; this study focused only T2DM patients.

**Answer:**

Thank you for your great comment.

We’ve added the analysis of the association between MUAC and glycemic parameters in the RESULTS, table 1, table 2 and table3 (see Paragraph 2 in Page 8 of the resubmitted manuscript with tracked changes). However, the association between MUAC and glycemic parameters was not observed in our research.

We acknowledged that Hou’s study also focused on MUAC in the Chinese population. However, the study was different from ours. Firstly, the population in their study included both participates with diabetes or normal glucose tolerance (NGT). The analysis of association between MUAC and central obesity or IR was conducted in all the participants but not just in patients with diabetes. The only analysis specially involved with diabetes was the multivariable logistic regression analysis base on the subgroups of diabetes or not. The key points that two studies concerned were different. As the importance of IR in diabetes was reinforced in the most recent studies^1-2^ and the central obesity, IR and MUAC in diabetes were quite different from those in participants with NGTs^3^, the conclusion and significance of Hou’s study could not be drawn to the population with diabetes. Secondly, the aim of Hou’s study was to investigate the associations between MUAC and cardiometabolic risk profiles but not mainly the MUAC and central obesity and IR.

The relevant contents were added in the INTRODUCTION (see Paragraph 1 in Page 4 of the resubmitted manuscript with tracked changes) and DISCUSSION (see Paragraph 2 in Page 17 of the resubmitted manuscript with tracked changes).

We acknowledged that the sample size in our research was relatively small. However, this is just a pilot study evaluating the association between MUAC and central obesity and IR specially in patients with diabetes. With the suggestive findings from this study, prospectively designed studies with more participants would be conducted in the near future. The relevant contents were added in the DISCUSSION (see Paragraph 3 in Page 18 of the resubmitted manuscript with tracked changes).

**[1] AHLQVIST E, STORM P, KARAJAMAKI A, et al. 2018. Novel subgroups of adult-onset diabetes and their association with outcomes: a data-driven cluster analysis of six variables[J]. Lancet Diabetes Endocrinol, 6(5): 361-369.**

**[2] ZOU X, ZHOU X, ZHU Z, et al. 2019. Novel subgroups of patients with adult-onset diabetes in Chinese and US populations[J]. Lancet Diabetes Endocrinol, 7(1): 9-11.**

**[3] YANG Q, GRAHAM T E, MODY N, et al. 2005. Serum retinol binding protein 4 contributes to insulin resistance in obesity and type 2 diabetes[J]. Nature, 436(7049): 356-362.**

**3、**In addition, medications of HT can also affect the results. Moreover, if patients were medicated by agents against hyperlipidemia and hyperuricemia, these must affect the results definitely. Authors should also investigate and mention them.

**Answer:**

Thank you for your comment.

We have added the details of the medications of hypertension, hyperlipidemia and hyperuricemia in the supplemental table 2, table3 and table4, respectively.

4、 Authors should explain log HOMAIR were used to investigation of association with MUAC. I would like to know why authors did not use HOMAIR itself. I would like to know whether authors considered statistical problem with using HOMAIR itself or not.

**Answer**:

Thank you for your comment.

As we mentioned in the Statistical analysis (see Paragraph 1 in Page 7 of the resubmitted manuscript with tracked changes), data of non-normal distributions were logarithmically transformed before statistical analysis.

As a result, to ensure normality of distribution and to meet the criteria for regression analysis, we used log HOMAIR to investigate the association of HOMAIR and MUAC as many other studies did^1-3^.

**[1] SANTHANAM P, ROWE S P, DIAS J P, et al. 2019. Relationship between DXA measured metrics of adiposity and glucose homeostasis; An analysis of the NHANES data[J]. PLoS One, 14(5): e0216900..**

**[2] MENTE A, MEYRE D, LANKTREE M B, et al. 2013. Causal relationship between adiponectin and metabolic traits: a Mendelian randomization study in a multiethnic population[J]. PLoS One, 8(6): e66808.**

**[3] ISHIMURA S, FURUHASHI M, WATANABE Y, et al. 2013. Circulating levels of fatty acid-binding protein family and metabolic phenotype in the general population[J]. PLoS One, 8(11): e81318.**

**Reviewer #1:**

1、This paper investigated the utility of measuring mid-upper arm circumference (MUAC) as a surrogate for measuring waist circumference or waist to hips ratio when assessing insulin resistance in patients with type 2 diabetes. It is concluded that MUAC is a simple tool to measure central obesity and insulin resistance. It is of interest that while MUAC correlated with BMI, waist circumference and and LDL-C in both genders, It failed to correlate with WHR, Tg, Uric acid, and HDL cholesterol in women. The implication of this failure in women was not discussed adequately. The possible reason (women are pear or apple shape while men are always apple shape) and possible consequence of using the MUAC as a clinical tool in women was not discussed.

**Answer:**

Thank you for your excellent comment.

It is interesting that while MUAC correlated with BMI, waist circumference and and LDL-C in both genders, but the correlation betwee MUAC and WHR, Tg, Uric acid, and HDL cholesterol in women was not observed. The plausible reason for this sex difference may be due to the different fat distribution during aging. Men have consistent fat distribution during aging, which is always characterized with more visceral fat in the abdomen (apple shape), but women have more subcutaneous fat in the hip and thighs(pear shape) before menopause and have more visceral fat in abdomen (apple shape) after menopause due to the dramatical decline of estrogen^1^. The female patients in our study, however, were mainly during the menopause period. As a result, no correlation was shown between MUAC and WHR in these estrogen-changing women. The differences of Tg, Uric acid, and HDL cholesterol also attributed to the decline of estrogen after menopause for the protective role of estrogen for metabolic diseases^2^. Therefore, using the MUAC as a clinical tool to detect some metabolic risk factors, such as WHR, Tg, Uric acid, and HDL cholesterol in women should be cautious.

The relevant contents were added in the DISCUSSION (see Paragraph 2 in Page 16 and Paragraph 1 in Page 17 of the resubmitted manuscript with tracked changes).

**[1] KARASTERGIOU K, SMITH S R, GREENBERG A S, et al. 2012. Sex differences in human adipose tissues - the biology of pear shape[J]. Biol Sex Differ, 3(1): 13.**

**[2] TRAMUNT B, SMATI S, GRANDGEORGE N, et al. 2019. Sex differences in metabolic regulation and diabetes susceptibility[J]. Diabetologia.**

2、On Page 19 Line 3 it is stated that "Furthermore we found MUAC might be superior to WC in measuring IR in type 2 diabetes..." It would be helpful to the reader to quote here the data and statistical result that support this statement.

**Answer:**

Thank you for your comment.

We’ve added the data and statistical result on 16 Page Line 1.

3、Minor Points

Page 4 Para 2 Line 3 "...it remains a number of limitations od WC.." should be "....there remain a number of limitations of WC.."

Page 4 Para 2 Line 11 "Therefore some Researches were conducted....." should be "Therefore som research was conducted..."

Page 4 Para 2 Line 15 "However it remains little data to..." should be "However there is little data to..."

There are many other examples where a native English speaker might help.

**Answer:**

We are sincerely sorry about the language issue. We have revised some mistakes as you suggested.

We’ll seek help for English editing company and thoroughly review and revise the manuscript in the next revision.
