# Supplementary material for: Mid-upper arm circumference as a simple tool for identifying central obesity and insulin resistance in type 2 diabetes
Source: PLoS One. 2020 May 21;15(5):e0231308. doi: 10.1371/journal.pone.0231308 (PMC7241705; doi:10.1371/journal.pone.0231308)
Supplement: Attachment — Submitted filename: Supplemental file for review .docx [file pone.0231308.s007.docx]

**Point-by –Point Answers to**

**the Editor’s and Reviewers’ comments**

Reviewer #2: Dear authors,

I think authors reviewed and answered against my comment carefully. Meanwhile, there are some remaining important problems, especially in the data authors added.
I commented to authors about the novelty of this study considering the previous article with large numbers. Actually I understand there is the difference between this and the previous article with regard to the subjects (the subjects of this study were only patients of T2DM). However, partly because this study cohort were not large, I commented to authors about various points.

At first, authors should consider the effect to results by the administration of subjects more. The supplemental table which authors added showed the difficulty of proving correctness of authors’ investigation and conclusion. I wrote that almost all agents for T2DM affected to HOMA-IR, especially not only sulphonylurea/glinide but also DPP4i /SGLT2i affected to insulin profile itself previously. Actually, authors could not remove the possibility of effect of anti-diabetic agents to the results and analysis of HOMA-IR. I even think it is possible diuretics or ARB/ACEi administration affected HOMA-IR.
Considering authors’ opinion of the novelty of this study (the subjects of this study were only patients of T2DM), authors must remove the possibility of effect of anti-diabetic agents at least.
Same as above, authors should reconsider table 6 and related results. LDL-C must be affected by statin. 70 of 103 patients were with statin in this study. Authors must consider the effect of statin to the results and analysis.
Honestly, I think all subjects should be without anti-diabetic agents. Furthermore, if authors would like to mention about the relationships including LDL-C, the subjects should be without agents against hyperlipidemia or authors should investigate and analyze considering the effect of agents against hyperlipidemia.
I would like authors to understand my previous and present comments adequately.
Authors’ revision considering these important points must be needed for this article to be accepted.

Regards,

**Answer:**

Thank you so much for your excellent comments.

First，honestly, we were very clear that all the pharmacotherapy of T2DM, hyperlipidemia or hypertention could definitely influenced insulin resistance. However, this was a cross-sectional study but not a case-control clinical trial, we could not intervene in any way to stop the anti-diabetic agents or any other medications as clinical trials do **^[1-2]^**. We can also see pharmacotherapy of patients with T2DM, hyperlipidemia or hypertention were not stopped in many similar studies involving the investigation of insulin resistance **^[3-5]^**.

Second, we should admit that we could not completely eliminate all the influencing factors. For example, if we stopped the pharmacotherapy before evaluating HOMA-IR (note that the mean diabetes duration of patients in our study was 8~9 years and the mean HbA1c was 9%), the plasma glucose would elevate, sometimes to a very high level. As is well known, the strongest insulin secretion influencing factor is glucose **^[6]^**. As a result, the elevating glucose would definitely influence the levels of insulin, and consequently influence HOMA-IR. Therefore, stopping the pharmacotherapy will still influence HOMA-IR.

Besides, in the guidelines of American Diabetes Association (ADA), European Association for the Study of Diabetes (EASD) or Chinese Diabetes Society (CDS), lifestyle intervention was recommended throughout the management of T2DM as a fundamental treatment **^[7-8].^** The lifestyle management can also significantly change the insulin resistance **^[9]^**. However, subjects in studies with pharmacotherapy or not could not stop lifestyle management, which consequently could not avoid its effect on HOMA-IR.

That may be the reason why the real-world study, with more diverse settings but less intervention, is gaining more and more concern **^[10]^**.

Third, as you pointed out that it is possible diuretics or ARB/ACEi administration could affect HOMA-IR, it would be most appropriate to include completely drug-naïve subjects into our study. However, the subjects included in our study were mostly in their middle age. Sixty-eight percent of middle-age and elderly population in our society had at least one chronic disease**^[11]^**. In addition, the prevalence of hypertension and hyperlipidemia in patients with diabetes is 51.9% and 30.5% respectively. More than 50% diabetic patients had at least one chronic diabetes complication **^[12-13]^**. As a society with dramatically increased aging population, we can hardly include the subjects at the middle age without any other diseases or any other drugs.

For the reasons above, we can not eliminate all the affecting factors including the effects of drugs or lifestyle intervention whether the cohort is large or not. As we mentioned in the discussion “ this is just a pilot study evaluating the association between MUAC and central obesity and IR, specially in patients with diabetes. Therefore, with the suggestive findings from this study, prospectively designed studies with more participants would be conducted in the near future” (see Paragraph 3 in Page 18 of the resubmitted manuscript with tracked changes), prospectively designed studies with drug-naive participants would be conducted in the near future at your great suggestions.

For the question of LDL-C affected by statin, besides the reasons mentioned above, we also conducted a logistic regression to adjust for using statin or not and find that using statin or not did not affect the results (OR：3.023 and P：0.033). (see Paragraph 3 in Page 11 of the resubmitted manuscript with tracked changes)

**[1] SEDGWICK P. 2015. Bias in observational study designs: cross sectional studies[J]. Bmj, 350: h1286.**

**[2] Kesmodel, Ulrik, S. Cross‐sectional studies – what are they good for?[J]. Acta Obstetricia et Gynecologica Scandinavica: Official Publication of the Nordisk Forening for Obstetrik och Gynekologi, 2018.**

**[3] Mamtani M , Kulkarni H , Dyer T D , et al. Waist Circumference Independently Associates with the Risk of Insulin Resistance and Type 2 Diabetes in Mexican American Families[J]. PLOS ONE, 2013, 8.**

**[4] Lim J S , Choi Y J , Kim S K , et al. Optimal Waist Circumference Cutoff Value Based on Insulin Resistance and Visceral Obesity in Koreans with Type 2 Diabetes[J]. Diabetes & Metabolism Journal, 2015, 39(3).**

**[5] Elkeles R S , Godsland I F , Feher M D , et al. Coronary calcium measurement improves prediction of cardiovascular events in asymptomatic patients with type 2 diabetes: the PREDICT study[J]. European Heart Journal, 2008, 29(18):2244-2251.**

**[6]Jackson R , Rudelt C , Willaime J P . Effects of prolonged glucose infusion on insulin secretion, clearance, and action in normal subjects.[J]. American Journal of Physiology, 1996, 270(2 Pt 1):E251**

**[7]2020. 6. Glycemic Targets: Standards of Medical Care in Diabetes-2020[J]. Diabetes Care, 43(Suppl 1): S66-s76.**

**[8]Bailey, Timothy. Options for Combination Therapy in Type 2 Diabetes: Comparison of the ADA/EASD Position Statement and AACE/ACE Algorithm[J]. The American Journal of Medicine, 2013, 126(9):S10-S20.**

**[9]SAMPATH KUMAR A, MAIYA A G, SHASTRY B A, et al. 2019. Exercise and insulin resistance in type 2 diabetes mellitus: A systematic review and meta-analysis[J]. Ann Phys Rehabil Med, 62(2): 98-103.**

**[10] Sherman R E , Anderson S A , Dal Pan G J , et al. Real-World Evidence — What Is It and What Can It Tell Us?[J]. New England Journal of Medicine, 2016, 375(23):2293-2297.**

**[11] Cheng Y，Cao Z，Hou J , et al. Investigation and association analysis of multimorbidity in middle-aged and elderly population in China, 2019, 23( 6):625-629.**

**[12] Ji L , Hu D , Pan C , et al. Primacy of the 3B Approach to Control Risk Factors for Cardiovascular Disease in Type 2 Diabetes Patients[J]. The American Journal of Medicine, 2013, 126(10):925.e11-925.e22.**

**[13] Liu Z , Fu C , Wang W , et al. Prevalence of chronic complications of type 2 diabetes mellitus in outpatients - a cross-sectional hospital based survey in urban China[J]. Health & Quality of Life Outcomes, 2010, 8(1):62-0.**

Reviewer #1: Thank you for addressing my concerns. Therefore it is now suitable for publication. I don't know if raw data has been made available.

**Answer:**

Thank you for your comment.

Raw data has been available now.
