# Supplementary material for: Mid-upper arm circumference as a simple tool for identifying central obesity and insulin resistance in type 2 diabetes
Source: PLoS One. 2020 May 21;15(5):e0231308. doi: 10.1371/journal.pone.0231308 (PMC7241705; doi:10.1371/journal.pone.0231308)
Supplement: Attachment — Submitted filename: Supplemental file for review.doc [file pone.0231308.s008.doc]

**Point-by –Point Answers to**

**the Editor’s and Reviewers’ comments**

Reviewer #2: Dear authors,

I am glad to see authors careful and proper answers to my comment.　
Firstly, authors performed modified logistic regression (adjusting for using statin or not) adequately. This must be needed and I am really glad to see this result.
In addition, authors wrote “this is just a pilot study evaluating the association between MUAC and central obesity and IR, specially in patients with diabetes” in discussion. I have been thinking authors should mention this, and authors did in this revised document. I am also glad to see the description.
Regarding effect of antidiabetic agents to results, I understand authors’ reply. As authors wrote in answers to my comments, this study was cross-sectional study. I think the study cohort was small as cross-sectional study. Of course, I understood anti-diabetic agents (as well as anti-hypertensive agents) should not be stopped in patients of T2DM. On the other hand, the effect to results should be considered because of this small cohort especially.
Considering above, authors should mention the limitation of this study more specifically. In detail, authors should add the description about the possibility of effect of anti-diabetic agents and anti-hypertensive agents to results, same as authors wrote in answers to my comments. Limitations should be written adequately in order to be understood by readers properly.
The revision of this article must be meaningful for authors as well as readers of this article. I really think authors considered my comments devotedly and modified properly. This must be the last recommendation to authors. I am looking forward to seeing authors’ revised document considering my comments.

[additional]
The description which I mentioned above, “this is just a pilot study evaluating the association between MUAC and central obesity and IR, specially in patients with diabetes”, should be modified to “this is just a pilot study evaluating the association between MUAC and central obesity and IR, “especially” in patients with “type 2 diabetes””. Same as this, the other descriptions, “patients with diabetes”, should be modified to “patients with type 2 diabetes”.

Sincerely,

**Answer:**

Thank you for your comments.

1. We have added the limitation of the effects of anti-diabetic agents and anti-hypertensive agents on our results in Discussion.(see Paragraph 1 in Page 19 of the resubmitted manuscript with tracked changes)
2. We have revised some mistakes as you suggested in [additional].

Regards.
